# Contrasting pathophysiological mechanisms of OPA1 mutations in autosomal dominant optic atrophy

**DOI:** 10.1038/s41420-025-02442-8

**Published:** 2025-05-30

**Authors:** Shi-Qi Yao, Jia-Jian Liang, Hui Zhou, Shaoying Tan, Yingjie Cao, Chong-Bo Chen, Ciyan Xu, Ruixi Wang, Tai-Ping Li, Fang-Fang Zhao, Yun Wang, Han-Jie He, Dan Zhang, Meng Wang, Lifang Liu, Patrick Yu-Wai-Man, Shihui Wei, Ling-Ping Cen

**Affiliations:** 1 Joint Shantou International Eye Center of Shantou University and The Chinese University of Hong Kong, Shantou, Guangdong China; 2 Shantou University Medical College, Shantou, Guangdong China; 3 School of Optometry, The Hong Kong Polytechnic University, Kowloon, Hong Kong; 4 University of Science and Technology Hospital, Shenzhen, Guangdong China; 5 John van Geest Centre for Brain Repair and MRC Mitochondrial Biology Unit, Department of Clinical Neurosciences, University of Cambridge, Cambridge, UK; 6 Cambridge Eye Unit, Addenbrooke’s Hospital, Cambridge University Hospitals NHS Foundation Trust, Cambridge, UK; 7 Moorfields Eye Hospital NHS Foundation Trust, London, UK; 8 Institute of Ophthalmology, University College London, London, UK; 9 Department of Ophthalmology, Third Medical Center of Chinese PLA General Hospital, Beijing, China; 10 Guangdong Provincial Key Laboratory of Medical Immunology and Molecular Diagnostics, The First Dongguan Affiliated Hospital, School of Medical Technology, Guangdong Medical University, Dongguan, China; 11 Dongguan Guangming Ophthalmic Hospital, Dongguan, Guangdong China

**Keywords:** Mechanisms of disease, Optic nerve diseases

## Abstract

Autosomal dominant optic atrophy (ADOA) caused by mutations in the nuclear-encoded OPA1 gene result in the preferential loss of retinal ganglion cells (RGCs) and progressive optic nerve degeneration. The severity of ADOA can be highly variable. This study compared the pathophysiological consequences of the c.1034 G > A OPA1 missense mutation and the c.1305+2delGT OPA1 deletion. There was a significant correlation between the severity of visual loss and the extent of macular RGC loss as determined by optical coherence tomography imaging. In cells transfected with the c.1034 G > A mutant, the percentage of fragmented mitochondria was greater than 60% with cytochrome c (cyt c) overflow, and significantly elevated levels of reactive oxygen species (ROS) and apoptosis. In contrast, the c.1305+2delGT mutant caused mitochondrial fragmentation in ~ 20% of HeLa cells, resulting in less cyt c overflow and apoptosis. The extent of mitochondrial network fragmentation and apoptosis increased with decreasing WT OPA1 mRNA expression levels. The c.1034 G > A OPA1 missense mutation is likely to induce a dominant-negative effect compared with haploinsufficiency with the c.1305+2delGT OPA1 deletion. These contrasting pathophysiological mechanisms could influence disease severity in ADOA through their differential consequences on mitochondrial structure and function. The small drug molecule Paromomycin was able to rescue the mitochondrial fragmentation induced by the c.1034 G > A mutation, providing proof-of-concept for further therapeutic validation in ADOA.

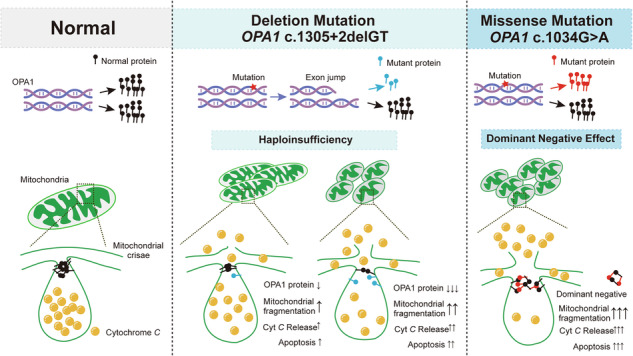

## Introduction

Autosomal dominant optic atrophy (ADOA; MIM#165500) is the most common inherited optic neuropathy with an estimated minimum frequency of 1:25,000 [1]. The disorder is characterized by insidious vision loss in early childhood with impaired color vision, a central or caecocentral scotoma, and temporal or diffuse pallor of the optic disc [2, 3]. Electrophysiologic and histopathologic studies are in keeping with the preferential loss of the retinal ganglion cells (RGCs), resulting in optic nerve degeneration and optic atrophy [4, 5].

More than five genes have been identified associated with ADOA, such as *OPA1, OPA3*, and *OPA4* [6–9]. Over 60% of ADOA cases are caused by mutations in the *OPA1* gene (3q28-q29), which spans over 60 kb of genomic DNA, and encodes for 30 exons and 8 isoforms generated by alternative splicing [1]. OPA1 is a multimeric GTPase protein associated with dynamin that localizes to the inner membrane of mitochondria [10]. OPA1 is essential for the maintenance of the morphology and function of mitochondria. This multimeric protein balances the fusion/fission processes within mitochondria to maintain mitochondrial network morphology [11, 12]. In addition, OPA1 has independent anti-apoptotic activity [13], as it tightens mitochondrial cristae junctions, isolating and preventing the release of pro-apoptotic cytochrome *c* (cyt *c*) molecules [14].

Over 600 potential variants have been reported in a site-specific database for *OPA1* (http://opa1.mitodyn.org/). More than 60% of these variants are considered pathogenic with two thirds located in the coding sequence. About 50% of the mutations result in premature truncation of OPA1 and nearly 40% result in loss of dynamin function. The majority of *OPA1* mutations are substitutions or deletions with only a few reported duplications, insertions, and in/del mutations [15]. Not all family members carrying pathogenic *OPA1* mutations develop visual impairment [16]. The lifetime probability of a mutation carrier becoming symptomatic has been estimated at 88% penetrance [17]. Moreover, there is marked intra and interfamilial variability in disease progression and manifestation, ranging from isolated optic atrophy to more severe multisystem involvement, referred to as ADOA plus [18]. This suggests that secondary factors are modulating disease expression in affected individuals.

In order to gain insight into the pathogenesis of ADOA and the factors influencing penetrance, we characterized three independent *OPA1* pathogenic mutations from three different families, examined their effects on mitochondrial morphology and cellular function in transfected HeLa and RGC5 cells, and further investigated the relationship between the *OPA1* mutations and disease penetrance and severity. Furthermore, we used Schrödinger’s high-throughput screening of small molecules to search for potential therapeutic agents for ADOA.

## Results

### Clinical Features of the Families Recruited with ADOA

**Fig. 1:**
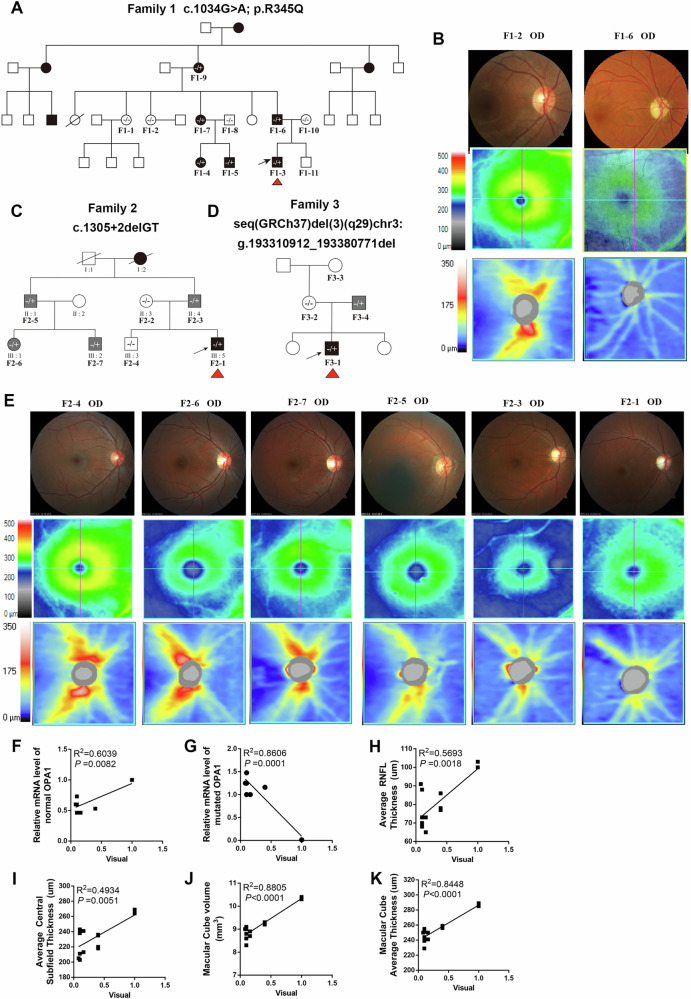
Clinical Features of ADOA Families. **A**, **C**, **D** Pedigree of the 3 families with ADOA indicating the segregation of the identified *OPA1* variants. Black symbols indicate patients with optic neuropathy; gray symbols indicate patients with subclinical disease. Arrows indicate family index cases. **B** Ophthalmologic features in family 1: an unaffected family member (F1-2) and a severely affected member (F1-6). **E** Ophthalmologic features in family 2: an unaffected family member (F2-4), four mildly affected family members (F2-6,7,5,3), and a severely affected family member (F2-1). **F**–**K** Correlation analysis between visual acuity and various parameters, including relative mRNA levels of normal and mutant *OPA1*, and optical coherence tomography measurements. The results showed a significant correlation. For each family member, the posterior fundus, RNFL thickness map and macular cube are shown. OD =“Oculus Dexter” or right eye; OS = “Oculus Sinister” or left eye.

Three families diagnosed with ADOA were recruited for this study. The proband of family 1 was initially seen at the age of 2 years old due to subnormal vision in both eyes but did not cooperate with visual acuity testing. There was a positive family history with other family members known to have poor vision. Fundus examination showed bilateral pale optic discs. There were 32 family members (19 males and 13 females), of which 10 was known to be visually affected (4 males and 6 females). A total of 10 individuals participated in the study, 6 affected and 4 unaffected family members (mean age = 28.9 years, SD = 20.4 years, range = 2-65 years) (Fig. 1A-B).

The proband of family 2 was assessed at the age of 17 years old with reduced visual acuity of 0.1 in the right eye and 0.08 in the left eye. Family 2 had a total of 10 family members (6 males and 4 females), including 6 affected individuals (4 males and 2 females). A total of 7 participants participated in the study, 5 affected and 2 unaffected family members (mean age = 32.7 years, SD = 14.9 years, range = 17–50 years) (Fig. 1C). Of the 5 affected family members, 3 were visually asymptomatic and they were found to have features of an optic neuropathy on examination.

The proband of family 3 was examined at the age of 5 years old with visual acuity of 0.1 in the right eye and 0.16 in the left eye. There was no known family history. His father who was found to have subclinical disease with bilateral optic atrophy was visually asymptomatic (Fig. 1D).

### Correlation Analysis between Visual Acuity and Fundus Changes

The available clinical data for individuals who were recruited from the three ADOA families was compiled (Table 1). Family 1 was fully penetrant with all *OPA1* mutation carriers being affected. In families 2 and 3, there were subclinically affected individuals who had a less severe phenotype with variable degrees of visual loss and changes on ophthalmological examination (Fig. 1E).

**Table 1:** Clinical data of individuals from the three families with ADOA included in this study.

Patient	Genotype	Gender	Age at inclusion (yr)	VA (OD/OS)	Average RNFL thickness(um) (OD/OS)	Average C/D Radio (OD/OS)	Vertical C/D Radio (OD/OS)	Cup Volume (mm2) (OD/OS)	Central Subfield Thickness(um) (OD/OS)	Macular Cube Volume(mm2) (OD/OS)	Macular Cube Average Thickness(um) (OD/OS)	Optic Disc Appearance
F1-2	WT	F	40	1.0/1.0	97/98	0.58/0.60	0.54/0.59	0.208/0.241	262/255	10.4/10.3	290/285	N
F1-5	c.1034 G > A	M	8	0.6/0.6	94/94	0.47/0.37	0.37/0.43	0.037/0.031	207/206	9.6/9.6	266/266	SP
F1-6	c.1034 G > A	M	32	0.01/0.01	55/56	0.66/0.77	0.71/0.71	0.136/0.273	239/234	9.1/8.8	254/246	MP
F1-9	c.1034 G > A	F	65	0.02/0.02	58/54	0.81/0.80	0.76/0.74	0.46/0.359	251/250	8.7/8.6	245/242	MP
F2-1	c.1305+2delGT	M	16	0.1/0.16	70/65	0.76/0.76	0.74/0.71	0.421/0.411	238/241	9.1/8.9	255/250	MP
F2-3	c.1305+2delGT	M	46	0.1/0.1	70/68	0.73/0.75	0.66/0.71	0.310/0.313	243/240	8.3/8.8	229/244	SP
F2-4	WT	M	18	1.0/1.0	100/103	0.57/0.58	0.47/0.51	0.161/0.161	264/269	10.4/10.3	289/285	N
F2-5	c.1305+2delGT	M	49	NMD	79/82	0.67/0.66	0.60/0.66	0.265/0.180	216/211	9.0/8.8	250/245	N
F2-6	c.1305+2delGT	F	24	0.08/0.1	91/88	0.59/0.63	0.54/0.60	0.205/0.266	205/203	9/9	250/251	SP
F2-7	c.1305+2delGT	M	22	0.4/0.4	78/77	0.64/0.58	0.52/0.49	0.181/0.095	236/235	9.3/9.3	259/258	N
F3-1	g.193310912_ 193380771del	M	5	0.1/0.16	73/73	0.36/0.38	0.27/0.35	0.034/0.04	211/213	8.6/8.7	239/241	MP
F3-4	g.193310912_ 193380771del	M	32	0.4/0.4	78/86	0.54/0.52	0.46/0.46	0.127/0.098	220/218	9.3/9.2	257/256	N

All fundus data listed in the table were used for inclusion in the correlation analysis. *WT* wild type, *M* male, *F* female, *VA* visual acuity, OD right eye, OS left eye, *NMD* non-measurable data, *N* normal, *SP* Subtle pallor, *MP* Marked pallor.

The visual acuity of recruited individuals in families II and III was correlated with the expression level of OPA1, average retinal nerve fiber layer (RNFL) thickness, average cup-to-disc (C/D) radio, vertical C/D radio, cup volume, central subfield thickness, macular cube volume, macular cube average thickness correlations. The expression level of OPA1, average RNFL thickness, central subfield thickness, macular cube volume and macular cube average thickness was significantly correlated with visual acuity (Fig. 1F–K), indicated that the decrease in visual acuity had a strong correlation with the expression level of OPA1 and the degree of macular damage.

### Genetic Analysis Reveals Mutations in *OPA1*

**Fig. 2:**
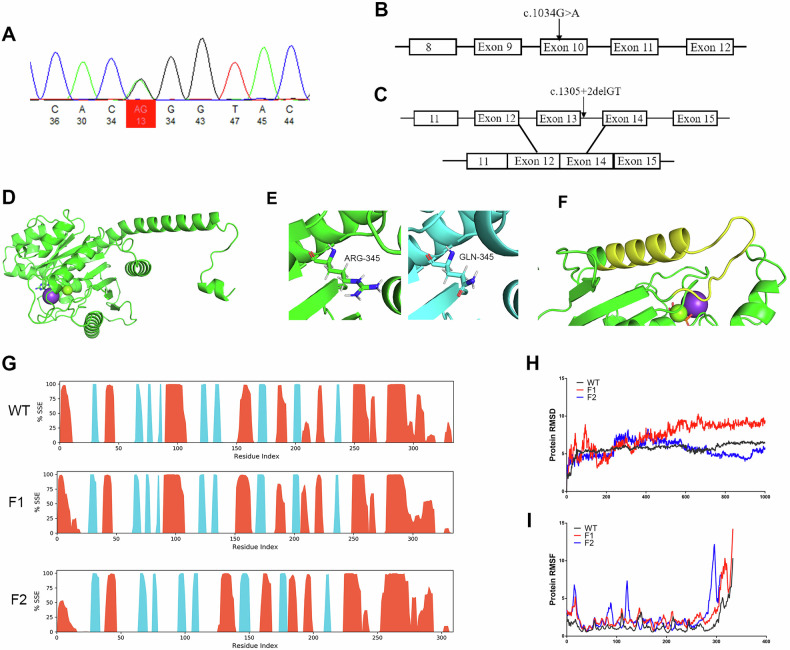
Genetic and Structure Analysis of *OPA1* Mutations. **A** Sequencing electropherograms showing the c.1034 G > A *OPA1* missense mutation in family 1. **B**, **C** Location of the c.1034 G > A *OPA1* missense mutation and c.1305+2delGT *OPA1* deletion and its impact on transcript processing. **D**–**F** The OPA1 protein structure was predicted using Alphafold2 derived for wild-type (WT) (**D**), c.1034 G > A (**E**), and c.1305+2delGT (**F**) transcripts. The amino acid residues that were predicted to be skipped as a result of the c.1305+2delGT *OPA1* deletion have been highlighted in yellow on the WT protein. **G** Protein secondary structure elements were monitored throughout the simulation with alpha-helices in orange and beta-strands in blue. **H, I** Stability and flexibility of the OPA1 proteins over the course of 100 ns. **H** Root mean square deviation (RMSD). **I** Root mean square fluctuation (RMSF).

Three different heterozygous *OPA1* mutations were detected by whole-exome sequencing in a total of 18 individuals in the three recruited ADOA families, namely, a missense mutation (c.1034 G > A: p.R345Q) (Fig. 2A, B), a splice-site deletion mutation (c.1305+2delGT) causing exon 13 skipping (Fig. 2C), and a deletion mutation (g.193310912_193380771del) of ~ 69.86 kilo base pairs (kbp) in the 3q29 region of the long arm of chromosome 3, which contains the *OPA1* gene. A web-based search revealed that mutations c.1305+2delGT and g.193310912_193380771del had not been previously reported. The c.1034 G > A and c.1305+2delGT mutations both affect highly conserved regions of the GTPase domain.

### Prediction of OPA1 Protein Stucture

The Alphafold2 platform was used to predict the structure of the mutant OPA1 protein. Although the missense c.1034 G > A mutation carried by family 1 caused the change of arginine at position 345 to glutamine (Fig. 2D-E), its secondary structure was not significantly altered (Fig. 2G). In contrast, the splice-site c.1305+2delGT deletion in family 2 (Fig. 2C) resulted in the loss of amino acids at positions 411-435 (Fig. 2F), affecting the normal secondary structure of the OPA1 protein (Fig. 2G).

### Molecular Dynamics Simulation of Protein Structure

The stability and structural flexibility of the WT and mutant proteins were investigated by root mean square deviation (RMSD) and root mean square fluctuation (RMSF), respectively, based on 100-ns MD simulations of the protein structures. The RMSD of the WT OPA1 protein reached 6.476 Å at 100 ns and the RMSD remained stable throughout the simulation. The c.1034 G > A mutant protein reached an RMSD of 9.433 Å at 100 ns, which was higher than the WT structure, but it fluctuated more than 5 Å during the simulation process. In contrast, the c.1305+2delGT mutant protein had an RMSD of 5.828 Å, which was lower than the WT structure, and it manifested fluctuations during the simulation process (Fig. 2H). These findings indicate that both *OPA1* mutations are likely to affect the protein stability of OPA1.

Simulations of protein structural flexibility showed four peaks in the c.1305+2delGT mutant with an RMSF value of more than 5 Å. These peaks indicate the most fluctuating regions of the protein. These findings suggest that the flexibility of these active-site residues may be affected making them structurally unstable, and potentially impairing the binding of OPA1 with other proteins. In contrast, the c.1034 G > A mutant and the WT protein were essentially identical in terms of RMSF trajectory (Fig. 2I), indicating that the c.1034 G > A mutation did not have an effect on the structural flexibility of the protein.

### *OPA1* c.1034 G > A Impairs Mitochondrial Morphology More Than c.1305+2delGT

**Fig. 3:**
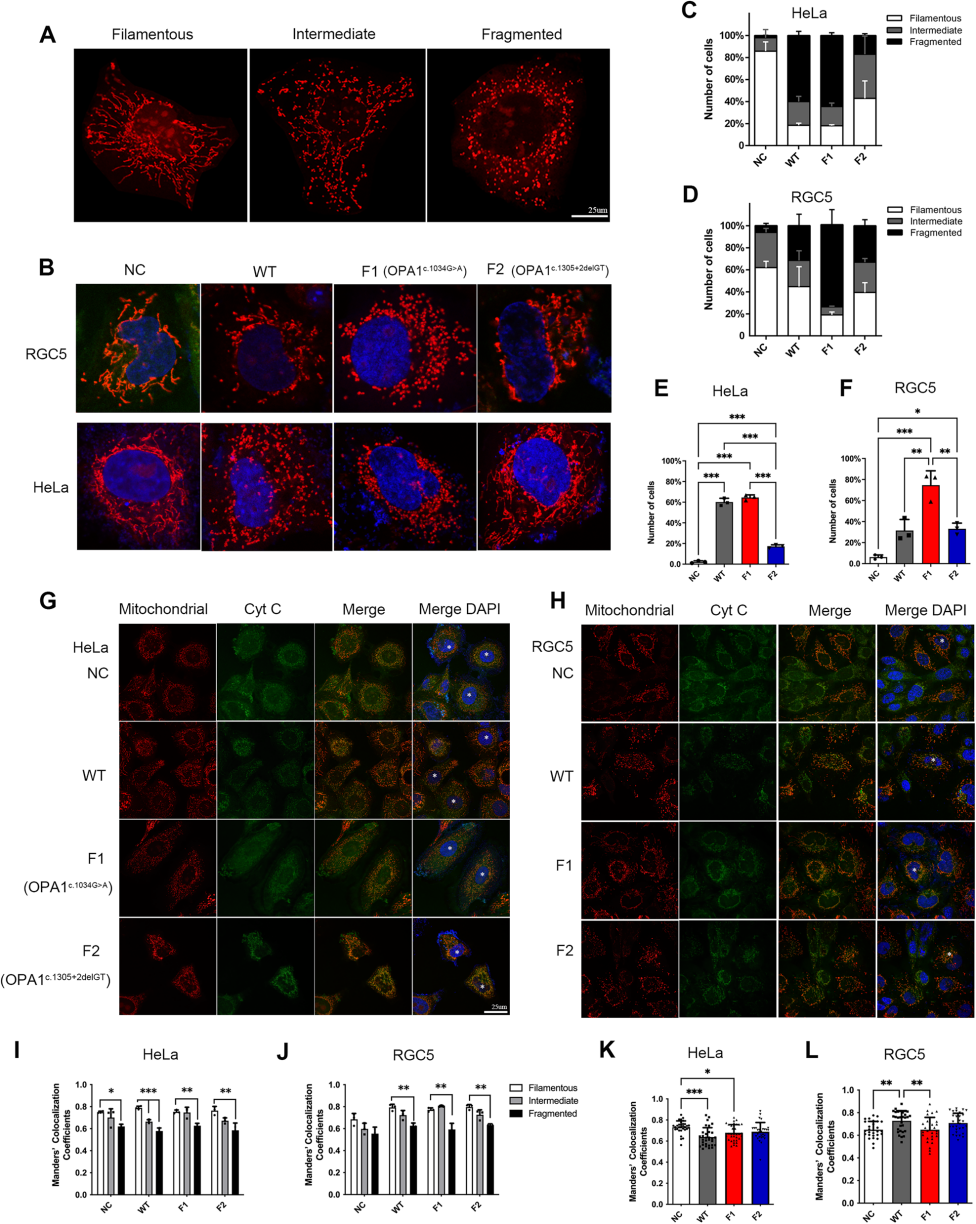
Mitochondrial Morphology and Function analysis of *OPA1* Mutations. **A, B** Mitochondrial morphology in HeLa and RGC5 cells overexpressing *OPA1* mutants. Mitochondria were marked by red fluorescence. The cell nucleus was stained by DAPI. **C**–**F** Quantitative analysis of mitochondrial morphology classified as filamentous (white), intermediate (gray), and fragmented (black). The HeLa and RGC5 cells transfected with the c.1034 G > A mutant displayed a fragmented mitochondrial network. **G, H** Colocalization between cytochrome (cyt) *c* (green fluorescence) and mitochondrial in HeLa and RGC5 cells overexpressing *OPA1* mutants. The transfected cells have been indicated with stars. **I**–**L** Quantitative analysis of colocalization coefficients of cyt c and mitochondria. The cells transfected with the c.1034 G > A mutant displayed a decreased coefficient. Asterisks indicate statistical significance (*adjusted *p* < 0.05, **adjusted *p* < 0.01, ***adjusted *p* < 0.001).

Three separate sets of plasmids containing blank vector, WT and pathogenic OPA1 alleles were transfected into HeLa and RGC5 cells for overexpression. The effect of these two *OPA1* mutations on the morphology of mitochondrial network was assessed, and categorized into filamentous, intermediate and fragmented mitochondria (Fig. 3A).

Overexpression of the c.1034 G > A mutant caused severe fragmentation of the mitochondrial network in both RGC5 and HeLa cells (Fig. 3B), with > 60% of cells exhibiting fragmented mitochondria (*P* < 0.001) (Fig. 3C). In contrast, overexpression of the c.1305+2delGT mutant caused mitochondrial fragmentation in ~ 20% of RGC5 (*P* < 0.05) and HeLa cells (*P* < 0.001) (Fig. 3C–F). Overexpression of wild-type OPA1 in HeLa and RGC5 cells also impaired normal mitochondrial morphology with mitochondrial network fragmentation observed in ~ 30% of RGC5 (*P* < 0.05) and ~ 60% of HeLa cells (*P* < 0.001), which is the same as previously reported [19].

*OPA1* mutations impair mitochondrial fusion changing the mitochondrial network from a filamentous to a fragmented distribution, with the effect being more marked with the c.1034 G > A mutant compared with the c.1305+2delGT mutant.

### *OPA1* c.1034 G > A Causes More Cytochrome *c* Overflow Than c.1305+2delGT

To further analyze the effect of *OPA1* mutation on mitochondrial function, we performed fluorescence co-localization analysis of cyt *c* and mitochondria (Fig. 3G, H). The analysis of different morphological mitochondrial networks showed that the co-localization coefficient of fragmented mitochondria was significantly lower than that of filamentous mitochondria (Fig. 3I, J).

Subsequent analysis of the cells as a whole showed that, compared with the control group, overexpression of the c.1034 G > A mutant in HeLa cells resulted in a significant decrease in the co-localization coefficient (Fig. 3K, L). Overexpression of the c.1305+2delGT mutant did not result in significant colocalization difference (Fig. 3K, L).

These findings indicated that cyt *c* was released from mitochondria into the cytoplasm after mitochondrial fragmentation. The release of cyt *c* caused by c.1034 G > A mutation was significantly compared with control and WT, while the release of cyt *c* caused by the c.1305+2delGT mutation was not different.

### OPA1 c.1034 G > A Induces More Apoptosis Than c.1305+2delGT

**Fig. 4:**
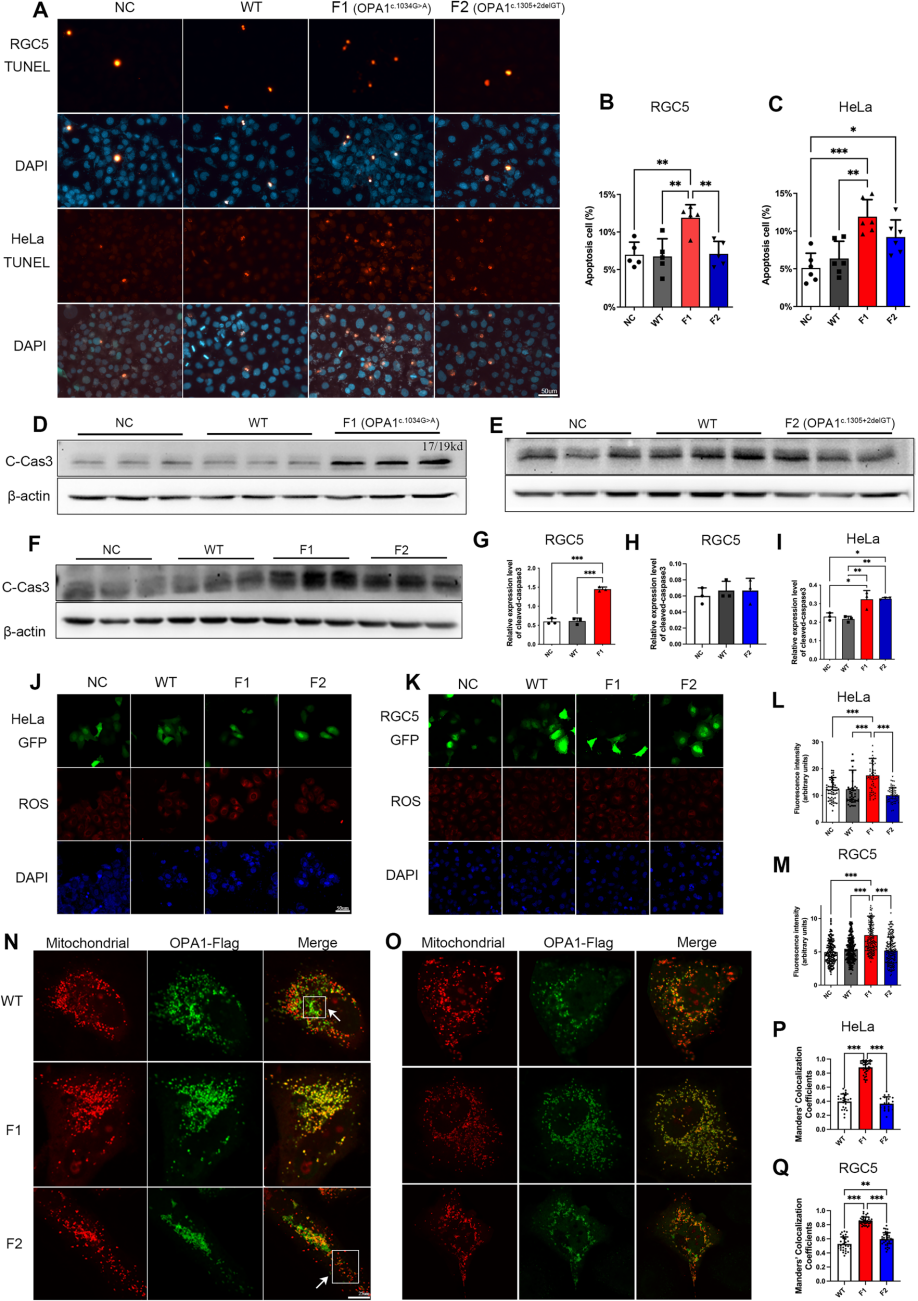
Apoptosis, ROS level and colocalization analysis of *OPA1* Mutations. **A** TUNEL assay of apoptosis in HeLa and RGC5 cells overexpressing *OPA1* mutants. **B**, **C** Quantitative analysis of the level of apoptosis. **D**–**F** Western blot of the cleaved-caspase3 expression level in HeLa and RGC5 cells overexpressing *OPA1* mutants. **G**–**I** Quantitative analysis of the level of apoptosis. Overexpression of c.1034 G > A mutant resulted in a significant increase in apoptosis. **J**, **K** Cellular reactive oxygen species (ROS) level in HeLa and RGC5 cells overexpressing *OPA1* mutants. **L**, **M** Quantitative analysis of the cellular ROS level. Both HeLa and RGC5 cells overexpressing *OPA1* c.1034 G > A had relatively higher cellular ROS levels. **N**, **O** Colocalization between *OPA1* mutants and fragmented mitochondrial in HeLa and RGC5 cells. The *OPA1* mutant was tagged by flag, shown in green fluorescence. **P**, **Q** Quantitative analysis of colocalization coefficients. *OPA1* c.1034 G > A was significantly colocalized with fragmented mitochondria.

TUNEL staining showed that overexpression of the c.1034 G > A mutant caused significant apoptosis with an increase in the percentage of apoptotic cells to 12% in both RGC5 (*P* < 0.01) and HeLa (*P* < 0.001) cells. Overexpression of the c.1305+2delGT mutant significantly increased the percentage of apoptotic cells to 9% in HeLa cells (*P* < 0.05), but it had no effect on apoptosis in RGC5 cells (Fig. 4A–C).

The WB results were consistent with the TUNEL results. Cleaved-caspase3 expression level was significantly increased in both HeLa (*P* < 0.05) and RGC5 (*P* < 0.001) overexpressing the c.1034 G > A mutant (Fig. 4D–I). In contrast, cleaved-caspase3 expression level of c.1305+2delGT mutant was significantly increased only in HeLa cells (*P* < 0.05) (Fig. 4I).

These results indicate that the c.1034 G > A mutation sensitized cells to apoptosis to a greater extent compared with the c.1305+2delGT mutation.

### *OPA1* c.1034 G > A Mutation Increases Reactive Oxygen Species Level

Overexpression of the c.1034 G > A mutant caused a significant increase in cellular ROS level in both HeLa and RGC5 cells (*P* < 0.001), whereas the c.1305+2delGT mutant had no significant effect (Fig. 4J-M).

These results showed that the more severe mitochondrial fragmentation and cyt *c* release caused by the c.1034 G > A mutation significantly increase cellular ROS level, whereas the c.1305+2delGT mutation did not impact mitochondrial morphology and function sufficiently to cause changes in cellular ROS level.

### Co-localization of *OPA1* c.1034 G > A Mutant and Fragmented Mitochondria Suggests a Dominant-Negative Effect

To further explore the relationship between OPA1 mutants and mitochondrial morphological and functional impairments, the fluorescence co-localization of fragmented mitochondria and *OPA1* mutants was analyzed (Fig. 4N-O). The cellular localization of *OPA1* mutants overlapped with mitochondria, which is consistent with OPA1 being a mitochondrial inner membrane protein, and this is not affected by *OPA1* mutations [20].

In both HeLa and RGC5 cells overexpressing the c.1034 G > A mutant, the colocalization coefficient of the c.1034 G > A mutant with fragmented mitochondria was significantly higher than that seen with the c.1305+2delGT mutant and WT alleles (*P* < 0.001) (Fig. 4P-Q). These findings suggest that the c.1034 G > A mutant could be exerting a more deleterious dominant-negative effect compared with the mechanism associated with the c.1305+2delGT mutant.

### The Expression Level of *OPA1* c.1305+2delGT Mutants Suggests Haploinsufficiency

**Fig. 5:**
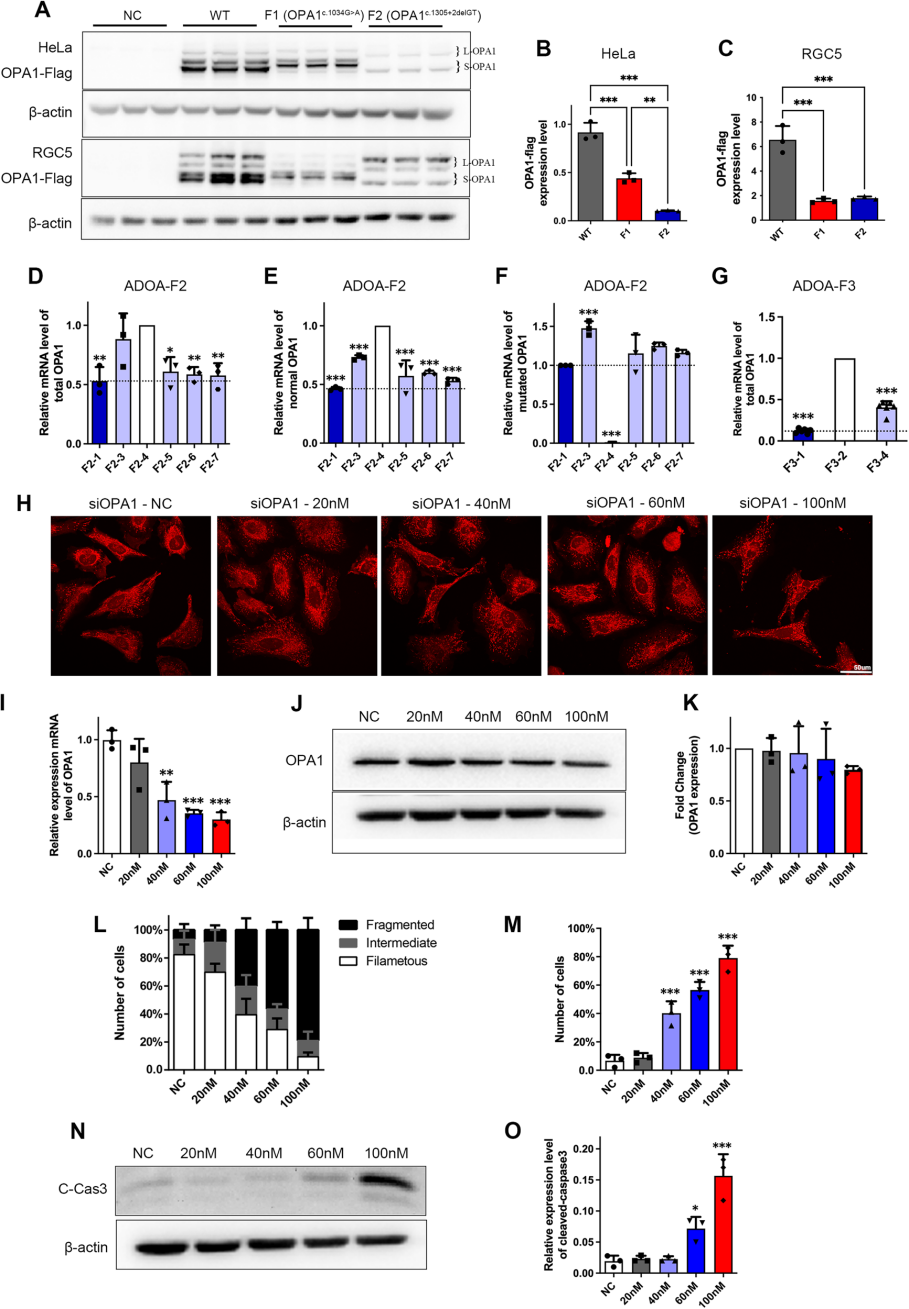
OPA1 Expression Level related analysis. **A** Western blot of the OPA1 isoforms and expression level in HeLa and RGC5 cells overexpressing different *OPA1* mutants. The two OPA1 isoforms were tagged by flag. **B**, **C** Quantitative analysis of the expression level of OPA1 mutant proteins. The expression level of OPA1 mutant proteins decreased significantly. **D**–**G** *OPA1* mRNA expression levels in blood samples from ADOA family members. Unaffected family members are show in white, mildly affected family members in light purple, and the proband in dark blue. **H** Mitochondrial morphology in HeLa cells transfected with different concentrations of *OPA1* siRNA. **I** *OPA1* mRNA expression level detection by qPCR. **J**, **K** Quantification of OPA1 with Western blot. **L**, **M** Quantitative analysis of mitochondrial morphology. Loss of OPA1 induced fragmentation of the mitochondrial network. **N**, **O** Western blot of the c-cas3 expression level in HeLa cells transfected with different concentrations of *OPA1* siRNA. Decreased OPA1 led to mitochondrial fragmentation with a significant increase in apoptosis.

In cells transfected with the c.1305+2delGT mutant, two OPA1 isoforms, long OPA1 (l-OPA1) and short OPA1 (s-OPA1), were identified by Western blot. In HeLa and RGC5 cells, the OPA1 c.1034 G > A protein showed a completely different isoform pattern, identified the remaining two s-OPA1 (Fig. 5A). The expression level of the OPA1 c.1305+2delGT protein was significantly reduced to only 11% of that of the WT OPA1 protein (*P* < 0.001) (Fig. 5B-C). The exon skipping caused by the c.1305+2delGT deletion is likely to have a major impact on OPA1 protein stability, with haploinsufficiency being the main pathogenic mechanism.

*OPA1* mRNA expression levels were significantly reduced in all family members carrying the c.1305+2delGT mutation, but to variable levels (*P* < 0.01) (Fig. 5D). The expression of the WT *OPA1* allele was lower than 50% in the proband, but it was higher than 50% in all the other family members with subclinical disease (Fig. 5E). The expression level of the mutant OPA1 allele in these family members was also higher than in the proband (Fig. 5F). These differences in WT OPA1 mRNA expression levels were also observed in members of the family 3 that carried the large genomic deletion in chromosome 3, g.193310912_193380771del (*P* < 0.001) (Fig. 5G). These findings indicate that disease severity in ADOA may be influenced by the level of expression of the WT *OPA1* allele.

### Mitochondrial Morphology and Cell Apoptosis were Associated with the Expression Level of WT *OPA1*

To further clarify the relationship between *OPA1* expression level and disease severity in ADOA, the impact of *OPA1* siRNAs in HeLa cells was compared with that of control siRNA (Fig. 5H). *OPA1* expression gradually decreased with increasing siRNA concentrations (Fig. 5I–K). When the *OPA1* level decreased by ~50%, 40% of the cells showed fragmented mitochondrial network (*P* < 0.001). When the OPA1 level decreased by ~70%, 80% of the cells showed fragmented mitochondrial network, and the filamentous phenotype was less than 10% (*P* < 0.001) (Fig. 5L, M), which confirmed the fragmentation of mitochondrial network caused by the loss of OPA1.

WB experiments showed that the gradual fragmentation of mitochondria was accompanied by a significant increase in apoptosis (*P* < 0.001) (Fig. 5N, O), confirming that mitochondrial fragmentation and apoptosis were associated with the normal expression level of *OPA1*.

### High-Throughput Drug Screening Showed Rescue of Mitochondrial Fragmentation with Paromomycin

**Fig. 6:**
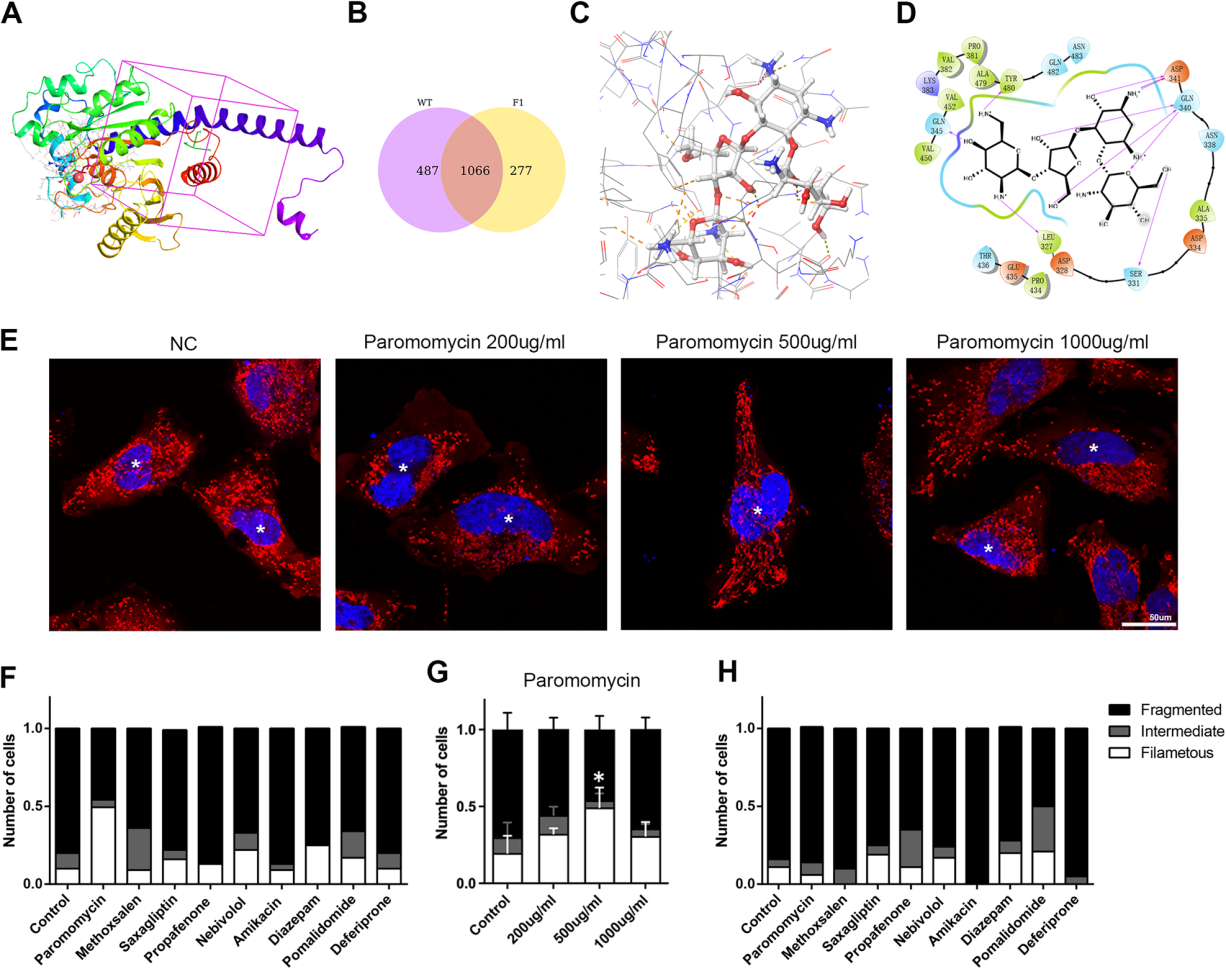
High-Throughput Screening for c.1034 G > A Mutant. **A** High-throughput screening of about 1,500 FDA-approved small drug molecules was performed using the GLIDE module of Schrödinger Suites centered on the *OPA1* missense mutation site. **B** Venn diagram showed the number of small molecules bound to WT and the OPA1 c.1034 G > A protein. **C**, **D** Molecular docking poses for paromomycin and OPA1 c.1034 G > A protein. **E** Mitochondrial morphology in HeLa cells overexpressing the c.1034 G > A mutant when treated with paromomycin. **F**–**H** Quantitative analysis of mitochondrial morphology. Paromomycin (500ug/ml) was able to resist the dominant-negative mechanism of the c.1034 G > A mutant by competitive binding.

In order to find drugs that could rescue the mitochondrial fragmentation caused by the dominant-negative effects of the c.1034 G > A mutant protein, we applied Schrödinger2021-1 to conduct a high-throughput screening of about 1,500 FDA-approved small molecule drugs (Fig. 6A). A total of 277 small molecule structures were able to bind to the c.1034 G > A mutant protein, but not to the WT protein (Fig. 6B). The 9 small molecule structures with the highest docking scores were selected for cellular experiments to observe their effects on mitochondrial morphology (Fig. 6C, D, Supplementary Table 1, Supplementary Fig 1-3).

The results showed that the addition of 500ug/ml Paromomycin along with the transfection of the c.1034 G > A plasmid resulted in a significantly higher proportion of filamentous mitochondria after 48 hours than that of HeLa cells transfected with c.1034 G > A plasmid only (Fig. 6E–G). In contrast, in HeLa cells transfected with the c.1034 G > A plasmid that were treated with Paromomycin 48 h later, the proportion of filamentous mitochondria did not change significantly (Fig. 6H). These results indicate that Paromomycin could block the dominant-negative mechanism of the c.1034 G > A mutant by competitive binding, thus preventing the fragmentation of the mitochondrial network.

## Discussion

In this study, we included three ADOA families carrying different pathogenic *OPA1* mutations. The c.1034 G > A missense mutation in family 1 has previously been reported to have dominant-negative effects [19]. Both the c.1305+2delGT deletion in family 2, which results in skipping of exon 13, and the large genomic deletion of chromosome 3 g.193310912_193380771del, which includes OPA1, have not been reported previously. The c.1034 G > A and c.1305+2delGT mutations are both located in a highly conserved region of the GTPase domain that is critical for OPA1-mediated mitochondrial fusion [21].

The c.1034 G > A mutant was more deleterious than the c.1305+2delGT mutant, resulting in greater mitochondrial fragmentation, unregulated cyt *c* release, a greater sensitivity to caspase-dependent apoptosis and elevated ROS levels. The dominant-negative role of the c.1034 G > A mutant was verified by the high degree of co-localization with fragmented mitochondria, which is the ability of the mutant dynamin to form oligomers with the WT protein, thereby interfering with its catalytic GTPase activity [22]. Consistent with previous report, overexpression of OPA1 WT in HeLa cells resulted in mitochondrial fragmentation [23]. This may be a non-specific effect due to overexpression of membrane proteins in the filamentous mitochondrial network [19]. There was no significant co-localization of the overexpressed OPA1 WT protein with fragmented mitochondria, implying that a dominant-negative effect is unlikely.

OPA1 dysfunction could result in ROS overproduction and redox homeostasis imbalance [15]. Unsurprisingly, we found elevated ROS levels in cells overexpressing the c.1034 G > A mutant. In a mouse model, heterozygous *OPA1* mutations led to elevated ROS levels and mitochondrial fragmentation [24]. Similarly, altered levels of antioxidant enzymes were found in fibroblasts established from patients with ADOA carrying *OPA1* mutations, further supporting the perturbation of the mitochondrial redox state [25]. Cellular stress response due to increased ROS could be an important contributor to the loss of RGCs and the progression of the optic neuropathy in ADOA.

In contrast to the c.1034 G > A mutant protein, the c.1305+2delGT mutant protein cannot integrate into oligomers to affect mitochondrial network dynamics in a dominant-negative manner. Our data showed that OPA1 c.1305+2delGT was rapidly degraded post-translationally in cellular models. The total amount of OPA1 in the blood cells of patients carrying this particular mutation was lower than in normal subjects, and the expression level of OPA1 varied among the patients. Thus, the exon skipping caused by the c.1305+2delGT deletion acts primarily through haploinsufficiency.

The OPA1 mutant may affect the protein stability and function by altering the expression of its isoforms. The c.1305+2delGT mutant exhibited different levels of OPA1, which may be due to the damage of protein folding and dimerization induced by the loss of exon 13, interfering with the processing of protein expression. Mutations in the GTPase region may directly affect the GTP hydrolysis activity of OPA1, lead to instability of L-OPA1 and make it more susceptible to cleavage [26]. In addition, cells may regulate the level and cleavage of OPA1 through feedback mechanisms to cope with the effects of impaired GTPase activity. Both the short and long forms of OPA1 are equally effective in maintaining cristae density and cristae junction tightness [27, 28]. Differences in OPA1 isoforms between the c.1034 G > A and c.1305+2delGT mutants could contribute to their differences in pathogenic mechanisms and disease severity.

The cells constructed in our study were overexpression models, which means that in addition to the mutant *OPA1* allele, the two WT alleles remained functional. Haploinsufficiency is therefore unable to exert its pathogenic role in the overexpression cell model, which could explain why the c.1305+2delGT mutant had no significant effect on cyt *c* release, ROS level, or apoptosis, although it triggered mitochondrial fragmentation to some extent. This overexpression model could also explain the differences in colocalization between OPA1 and fragmented mitochondria. c.1034 G > A OPA1 shows strong colocalization with mitochondria, suggesting that the binding of c.1034 G > A OPA1 to mitochondrial membrane may have a higher affinity than endogenous OPA1 in the cell. In contrast, overexpressed WT OPA1 and c.1305+2delGT OPA1 do not appear to exhibit a stronger binding affinity than endogenous OPA1 and therefore appear to be OPA1 proteins that are not colocalized with mitochondria. This difference in colocalization patterns further confirms the dominant negative effect of c.1034 G > A OPA1 on mitochondrial function.

In our study, disease severity with the c.1305+2delGT deletion correlated with the expression of WT OPA1. Total OPA1 and WT OPA1 were higher in family members with milder subclinical disease. If there is insufficient total OPA1, a cell could put in place compensatory mechanisms to stimulate the production of more OPA1 to fulfill its physiological functions. Based on siRNA modification experiments, the extent of mitochondrial fragmentation and apoptosis closely paralleled the reduction of OPA1 levels, thus confirming our observations in blood samples from ADOA families.

The factors influencing disease penetrance and severity in ADOA have not been fully elucidated and it is likely to be a complex system [29]. One possible explanation is that epigenetic modifications may compensate for the presence of deleterious variants [30]. This could potentially explain phenotypic differences between individuals carrying the same pathogenic genotype. Haploinsufficiency could also affect the expression of other genes in the same network to compensate the *OPA1* disease allele thus maintaining homeostasis [31]. This phenomenon has been demonstrated in model organisms [32] and, therefore, could be relevant in humans [33].

Finally, we have identified a possible therapeutic approach for *OPA1* missense mutations acting through a dominant-negative mechanism. Small drug molecules screened with mutant amino acids as docking sites can prevent mitochondrial fragmentation by competitive binding in cell culture. These are preliminary findings that will need to be confirmed in clinical trials to assess the visual benefit in affected individuals with ADOA. For *OPA1* mutations resulting in haploinsufficiency, gene replacement therapy or antisense oligonucleotide approaches to modulate mRNA expression are being considered [34].

In summary, the *OPA1* c.1034 G > A missense mutation in the structural GTPase domain is likely to be deleterious due to its dominant-negative effects on mitochondrial function, whereas the c.1305+2delGT deletion is causing disease through haploinsufficiency, which is consistent with previous studies [35, 36]. The small drug molecule Paromomycin, which was identified using high-throughput screening, was able to rescue the mitochondrial fragmentation induced by the c.1034 G > A mutation, providing proof-of-concept for further therapeutic validation in ADOA.

## Materials and Methods

### Experimental model and Subjects Details

A total of 20 individuals from 3 ADOA families were recruited from the Joint Shantou International Eye Center of Shantou University and The Chinese University of Hong Kong (JSIEC). The size of the pedigrees varied. The largest family spanned four generations that included ten assessed individuals. The diagnosis of ADOA and the criteria for inclusion in the study were bilateral optic nerve dysfunction with optic disc pallor and/or dyschromatopsia, and/or a positive family history. All included family members underwent a comprehensive ophthalmological examination, including fundus photography and optical coherence tomography imaging using the ZEISS platform (optic nerve and macula).

### Mutation Screening for the *OPA1* Gene

Genomic DNA was isolated from whole blood by using the TIANamp Genomic DNA Kit (Tiangen, Beijing) according to the manufacturer’s instruction. Whole exome capture and sequencing was performed by Novogene, Inc. (Beijing, China). All candidate pathogenic variants were confirmed by Sanger sequencing. We followed the standard nomenclature recommendations of the HGVS (http://www.HGVS.org/mutnomen/) to name the mutation(s). The mRNA sequence of *OPA1* isoform 8 (NM_130837) was used as the reference sequence.

### Protein Structure Prediction

Wild-type (WT) and mutant OPA1 protein structures were predicted with a protein structure prediction platform developed by our group based on the source code and trained models of Alphafold2, which can be accessed online [37]. Five structures were obtained for each input sequence. After performing an amber relaxation procedure on the unrelaxed structure prediction [38], these five models were ordered and ranked_0.pdb for the prediction. The one with the highest confidence was selected as the final model.

### Molecular Dynamics

Molecular Dynamics (MD) simulation was carried out by the Desmond module of the Schrödinger Suites (Schrödinger, Inc., USA, version 12.6). The predicted protein was used as the starting structure. Briefly, a model system was built using the system builder module in maestro, including determination of protonation states of residues after adding water box and counter ions (Na+ and Cl − ). TIP3P model was used for water molecules and the shape water box was set as orthorhombic. For all simulations, OPLS_2005 force field was used [39]. Finally, 100-ns simulation was carried out. The saved trajectories were analyzed using Simulation Interaction Diagram, including the root mean square deviation (RMSD) [40], root mean square fluctuation (RMSF).

### Plasmid Construction

The WT OPA1, c.1034 G > A and c.1305+2delGT mutant OPA1 plasmids were all constructed from Genechem, Inc. (Shanghai, China). PCR fragments were cloned in the GV208 vector, sequenced, and reintroduced into the pUbi- MCS-EGFP plasmid. The 3X flag tag was added to the C-terminus of the OPA1 protein for further detection.

### Cell Culture and Transfection

HeLa cells and RGC5 cells were grown in Dulbecco’s modified Eagle’s medium (DMEM; Gibco, USA) supplemented with 100 u/mL penicillin, 100 μg/mL streptomycin, and 10% Fetal Bovine Serum (FBS) at 37 °C with 5% CO2. When the cells had reached 80% confluency, a mixture of DNA and FuGENE HD transfection reagents (Promega, USA) was transfected in a 1:2 ratio. Cells transfected with empty expression vector were used as the controls. Cells were harvested at 48 h after the transfection.

Small drug molecules were solubilized according to the manufacturer’s instructions, and different concentrations were added to the medium at the same time as transfection or 48 hours after transfection, and incubation was continued for 48 hours after addition of the drugs.

### siRNA transfection

Silencing of the indicated genes was performed using forward transfection: 20 nM,40 nM,60 nM,100 nM of the specific siRNA was mixed with Lipofectamine RNAiMax (Invitrogen), added on HeLa cells, and left at 37 °C in a CO2 incubator for 72 h. Specific and non-targeting siRNAs were obtained from Obio Technology Co (Shanghai, China).

### Confocal Fluorescence Microscopy

Cells were cultured on collagen-coated cover glass slips (Marienfeld, Germany). CellROX® Deep Red Reagent was used for fluorescent labeling of cellular reactive oxygen species (ROS) level (Thermo Scientific, USA) at 5uM for 30 min at 37 °C, 5% CO2.

After three washes with PBS, cells were fixed with 4% paraformaldehyde for 15 min, then dissected, blocked and permeabilized with 5% normal goat serum (NGS) and 0.2% Triton X-100 for 30 min, incubated with the following antibodies for 2 h: Flag: 1/200 (Sigma-Aldrich, Germany); cyt *c*: 1/100 (Abcam, UK). After washing 3 times with phosphate-buffered saline (PBS), the cells were incubated with the respective secondary antibodies, Alexa-488 anti-rabbit IgG and Alexa-633 anti-rabbit IgG: 1/500), and stained with DAPI (1:500) for 1 h. After PBS washing, the coverslips were mounted on the slides with 50% glycerinum. Cells were examined using a confocal microscope (Leica TCS SP5 II).

### Western Blot

At 48 hours after transfection, HeLa cells and RGC5 cells were dissected and homogenized in cold radio immunoprecipitation (RIPA) lysis buffer supplemented with protease and phosphatase inhibitors. Total protein concentrations were evaluated by the Micro BCA Protein Assay Kit. After denaturating at 99 °C for 5 minutes, equal amount of total proteins (40 µg) in each samples were resolved using 10% or 15% SDS-PAGE and blotted onto the nitrocellulose membranes. The membranes were blocked in 5% non-fat milk solution and incubated with primary antibodies of OPA1 (Abcam, UK), flag (Yeasen, China), cleaved caspase3 (Cell signaling, USA) followed by respective horseradish peroxidase-conjugated secondary antibodies. The signal was visualized by enhanced chemiluminescence in ChemiDoc™ XRS^+^ system (Bio-Rad). The densitometry was determined and normalized to Beta-actin expression.

### Mitochondrial Network Analysis

Fluorescent labeling of mitochondria in live cells was achieved using MitoTracker® Orange-CMXRos (Thermo Scientific, USA) at 100 nM for 15 min at 37 °C, 5% CO2 and protect from light. The morphology of mitochondrial network was categorized into filamentous, intermediate and fragmented using the image processing package ImageJ (Fiji) [41].

### TUNEL analysis

Apoptosis was detected by TUNEL staining according to the manufacturer’s protocol (Roche, Switzerland). At 48 h after transfection, cells were fixed with 4% paraformaldehyde for 1 h, then blocked and permeabilized with 5% normal goat serum (NGS) and 0.2% Triton X-100 for 30 min, The coverslips were washed with PBS and incubated with TUNEL solution (450 μL label solution, 50 μL of enzyme solution) for 1 h at 37 °C in a moist and dark environment. The remaining 100 μL of label solution was used for negative controls for alternate sections. The coverslips were then washed with PBS. Subsequently, the coverslips were incubated with converter-POD solution in a humidified environment at 37 °C for 30 min. coverslips were then washed again with PBS. After washing, TUNEL positive cells were stained with diaminobenzidine tetrahydrochloride (DAB) as the chromogen.

### RT–qPCR

After white blood cells were isolated from whole blood samples using 1.8% NaCl solution, total RNA was extracted from the white blood cells and treated with RNase-free DNase I (Qiagen) according to the manufacturer’s protocol. Total RNA (0.5 μg) was reverse transcribed into cDNA using SuperScript III reverse transcriptase (Invitrogen). Gene expression was quantified with TB green PCR (Takara) in the LightCycle 480 II real-time PCR apparatus (Roche) with specific primers (Table S2) according to the manufacturer’s protocol. Relative gene expression was determined by the 2^−ΔΔCt^ method as compared to the negative control group and presented by the relative expression (ΔCt).

### High Throughput Virtual Screening

The ligand library used for high throughput virtual screening (HTVs) was from FDA-approved drugs ( ~ 1500 compounds) in the ZINC database (https://zinc.docking.org). After the preparation of protein and ligand library, amino acid position 345 of the mutant site was selected as the binding site. Ligand docking at this amino acid position was performed using the GLIDE module of Schrödinger Suites (Schrödinger, Inc., USA, version 12.6). High throughput virtual screening of the selected compound was performed against the target protein with a flexible docking parameter for compounds. After each compound was docked with the protein, the docking score is generated, and the affinity of the compounds was sorted according to the docking score.

### Quantification and statistical analysis

Data was presented as mean ± standard deviation (SD) (*n* = 3). After verifying whether the data followed a normal distribution and homogeneity of variance, one-way analysis of variance (ANOVA) with post-hoc Bonferroni test was applied to compare results of different groups. Independent T-test was used for the comparison between two groups. All statistical analyses were performed using a commercially available software (IBM SPSS Statistics 23; SPSS Inc., Chicago, IL). A P value of < 0.05 was considered as statistically significant.

## Supplementary information

Supplemental information

Supplementary Table 1

Supplementary Table 2.

Supplementary Figure 1

Supplementary Figure 2

Supplementary Figure 3

Original WB

## Data Availability

The datasets generated during and/or analysed during the current study are available from the corresponding author on reasonable request.
