# Supplementary material for: Contrasting pathophysiological mechanisms of OPA1 mutations in autosomal dominant optic atrophy
Source: Cell Death Discov. 2025 May 30;11:259. doi: 10.1038/s41420-025-02442-8 (PMC12125386; doi:10.1038/s41420-025-02442-8)
Supplement: Supplementary file 1 — Supplemental information [file 41420_2025_2442_MOESM1_ESM.docx]

**Supplemental information**

**Supplementary Figure 1.** Molecular docking poses for small molecules and the OPA1 c.1034G>A protein.

**Supplementary Figure 2.** Mitochondrial morphology in HeLa cells overexpressing the c.1034G>A mutant when treated with small molecules.

**Supplementary Figure 3.** Mitochondrial morphology in HeLa cells following treatment with small molecules 48 hours after the c.1034G>A mutant was overexpressed.

**Graph Abstract.** OPA1 is essential for the maintenance of mitochondrial morphology and function. OPA1 balances the internal mitochondrial fusion/fission process to maintain mitochondrial network morphology. In addition, OPA1 prevents apoptosis by forming homopolymers at mitochondrial cristae junctions, tightening these junctions and sequestering cytochrome *c*. The c.1305+2delGT *OPA1* deletion mutation results in varying degrees of apoptosis through a haploinsufficiency mechanism, with variable disease severity correlating with wild-type OPA1 expression level. In contrast, the c.1034G>A *OPA1* missense mutation has a more marked deleterious impact on mitochondrial morphology and function through a putative dominant-negative mechanism, resulting in more severe disease compared with the c.1305+2delGT deletion.
