# Supplementary material for: Contrasting pathophysiological mechanisms of OPA1 mutations in autosomal dominant optic atrophy
Source: Cell Death Discov. 2025 May 30;11:259. doi: 10.1038/s41420-025-02442-8 (PMC12125386; doi:10.1038/s41420-025-02442-8)
Supplement: Supplementary file 2 — Supplementary Table 1 [file 41420_2025_2442_MOESM2_ESM.docx]

**Supplementary Table 1. Details of the 9 small molecules with the highest docking score used in this study.**

|  | **Drug name (ZINC ID)** | **CAS NO** | **Formula** | **Molecular weight** | **Docking  score** |
| --- | --- | --- | --- | --- | --- |
| 1 | Paromomycin （ZINC60183170） | 1263-89-4 | C_23_H_47_N_5_O_18_S | 713.71 | -5.813 |
| 2 | Methoxsalen （ZINC2548959） | 298-81-7 | C_12_H_8_O_4_ | 216.19 | -5.717 |
| 3 | Saxagliptin （ZINC100037885） | 361442-04-8 | C_18_H_25_N_3_O_2_ | 315.41 | -5.703 |
| 4 | Propafenone （ZINC1530760） | 34183-22-7 | C_21_H_28_ClNO_3_ | 377.90 | -5.292 |
| 5 | Nebivolol （ZINC5844792） | 152520-56-4 | C_22_H_26_ClF_2_NO_4_ | 441.90 | -5.111 |
| 6 | Amikacin （ZINC8214483） | 39831-55-5 | C_22_H_47_N_5_O_21_S_2_ | 781.76 | -5.055 |
| 7 | Diazepam （ZINC6427） | 14439-61-3 | C_16_H_12_Cl_2_N_2_O | 319.19 | -4.842 |
| 8 | Pomalidomide （ZINC1997125） | 19171-19-8 | C_13_H_11_N_3_O_4_ | 273.24 | -4.679 |
| 9 | Deferiprone （ZINC6226） | 30652-11-0 | C_7_H_9_NO_2_ | 139.15 | -4.614 |
