# Supplementary material for: Contrasting pathophysiological mechanisms of OPA1 mutations in autosomal dominant optic atrophy
Source: Cell Death Discov. 2025 May 30;11:259. doi: 10.1038/s41420-025-02442-8 (PMC12125386; doi:10.1038/s41420-025-02442-8)
Supplement: Supplementary file 3 — Supplementary Table 2. [file 41420_2025_2442_MOESM3_ESM.docx]

| Gene | Forward primer (5‘-3’) | Reverse primer (5‘-3’) |
| --- | --- | --- |
| Total OPA1 | GACACGTTCTCCAGTTAAGGTGA | ATGGTCTCAGGGCTAACGGT |
| Normal OPA1 | GCTGTACCGTTAGCCCTGAG | CTTTGTGTCAGGAGCCATGC |
| Mutated OPA1 | CCCAAGAGGATCTGGGGAGA | AGGATATGGTATCTTCTTCTTTGGT |
| ACTB | CCCTGGACTTCGAGCAAGAG | ACTCCATGCCCAGGAAGGAA |
| OPA1 siRNA | AGACUAGUGUGUUGGAAAUTT | AUUUCCAACACACUAGUCUTT |
| siRNA-NTC | UUCUCCGAACGUGUCACGUTT | ACGUGACACGUUCGGAGAATT |
| siRNA-GAPDH | GUAUGACAACAGCCUCAAGTT | CUUGAGGCUGUUGUCAUACTT |

**Supplementary Table 2. The sequence of the primers and siRNA used in the study.**
