# Supplementary figures and images for: Contrasting pathophysiological mechanisms of OPA1 mutations in autosomal dominant optic atrophy
Source: Cell Death Discov. 2025 May 30;11:259. doi: 10.1038/s41420-025-02442-8 (PMC12125386; doi:10.1038/s41420-025-02442-8)

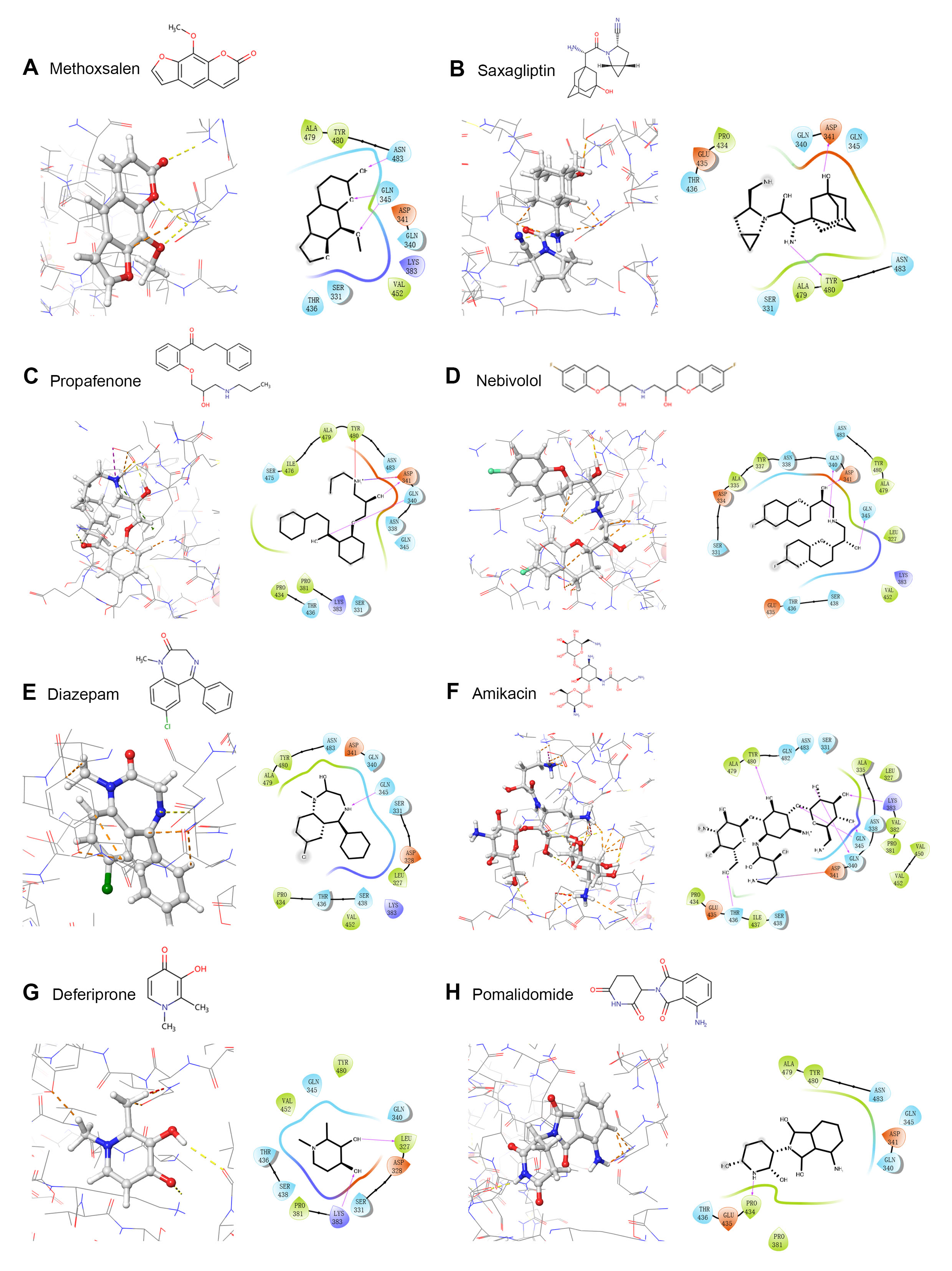

Supplement: Supplementary file 4 — Supplementary Figure 1 [file 41420_2025_2442_MOESM4_ESM.jpg]

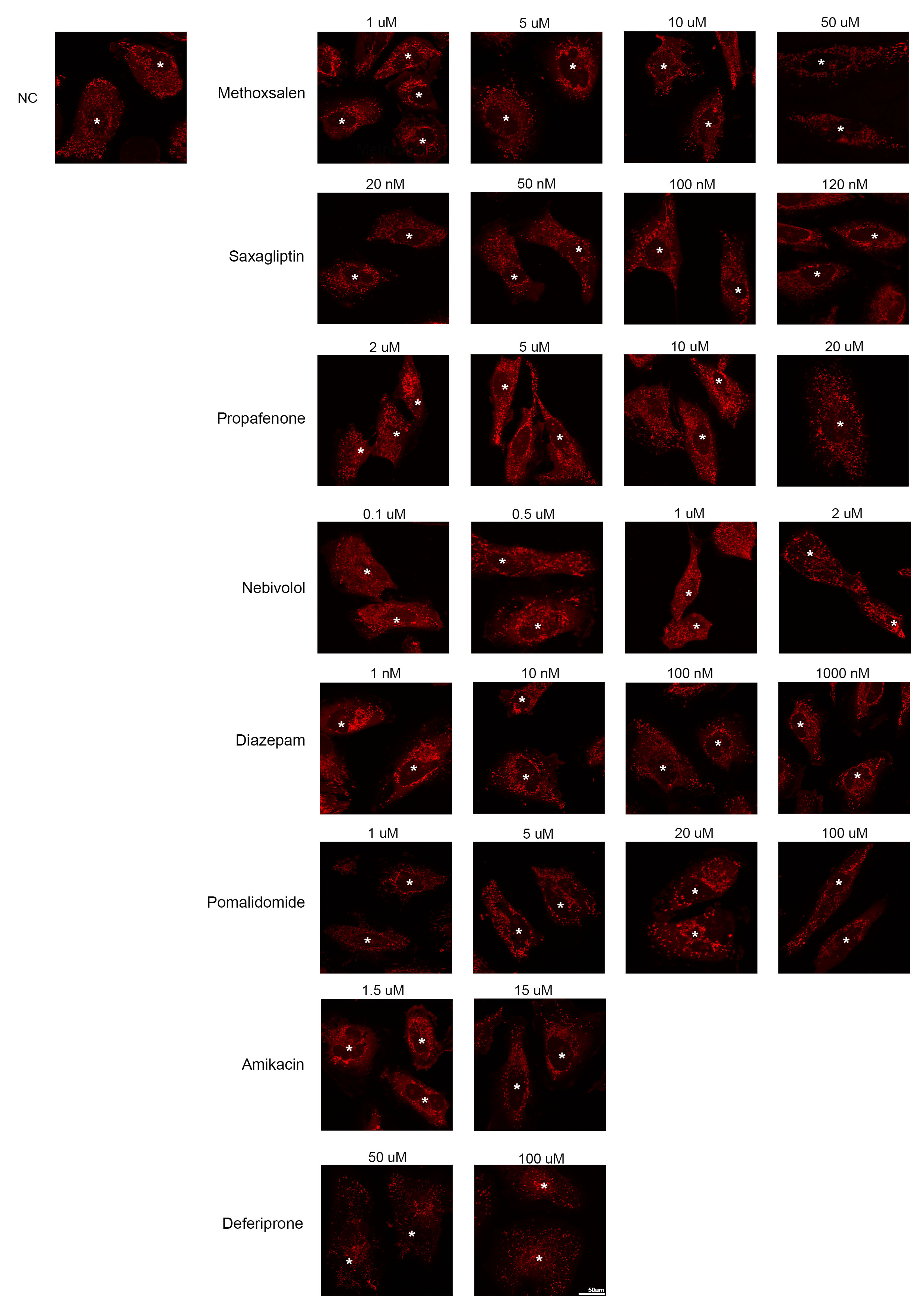

Supplement: Supplementary file 5 — Supplementary Figure 2 [file 41420_2025_2442_MOESM5_ESM.jpg]

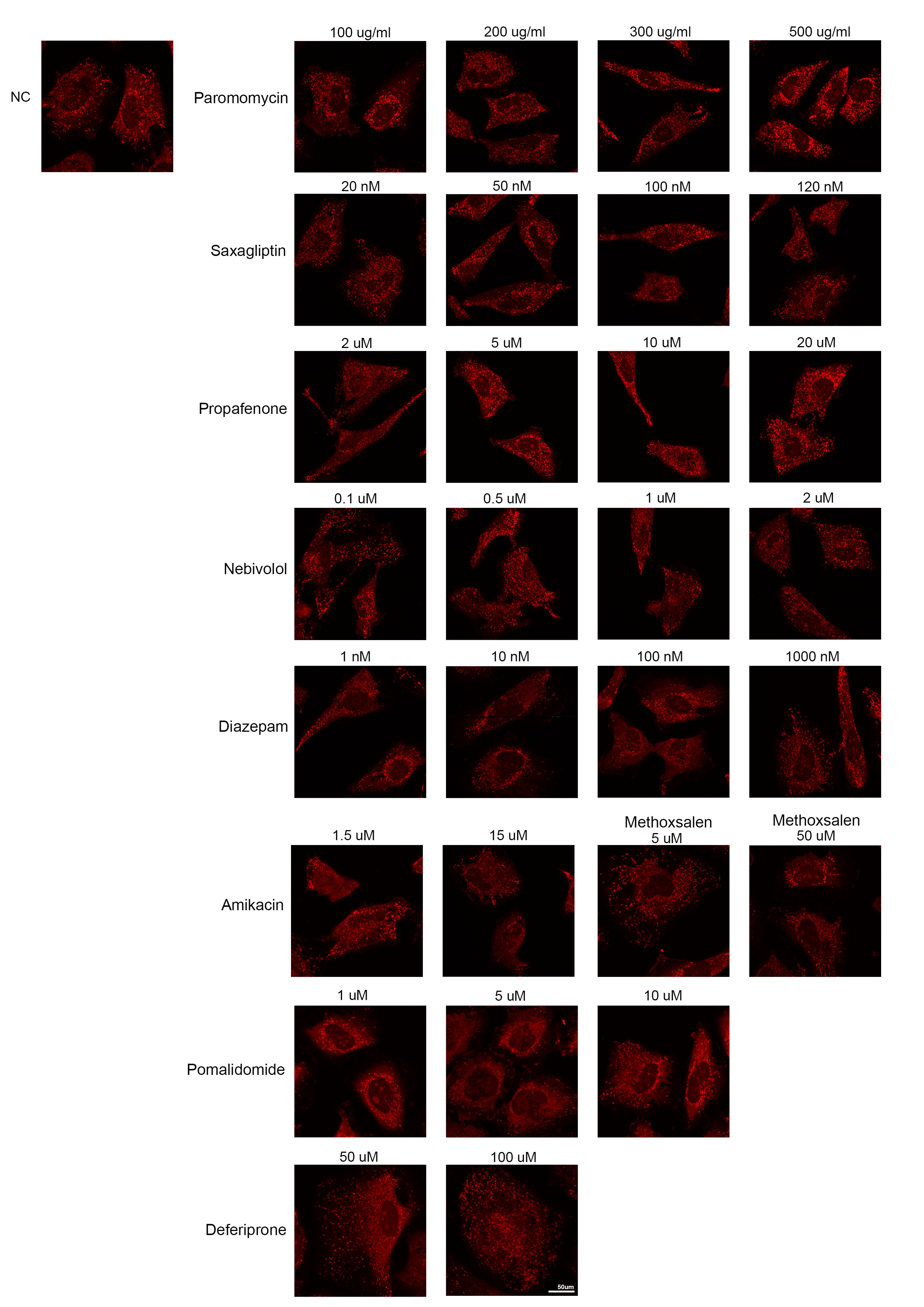

Supplement: Supplementary file 6 — Supplementary Figure 3 [file 41420_2025_2442_MOESM6_ESM.jpg]

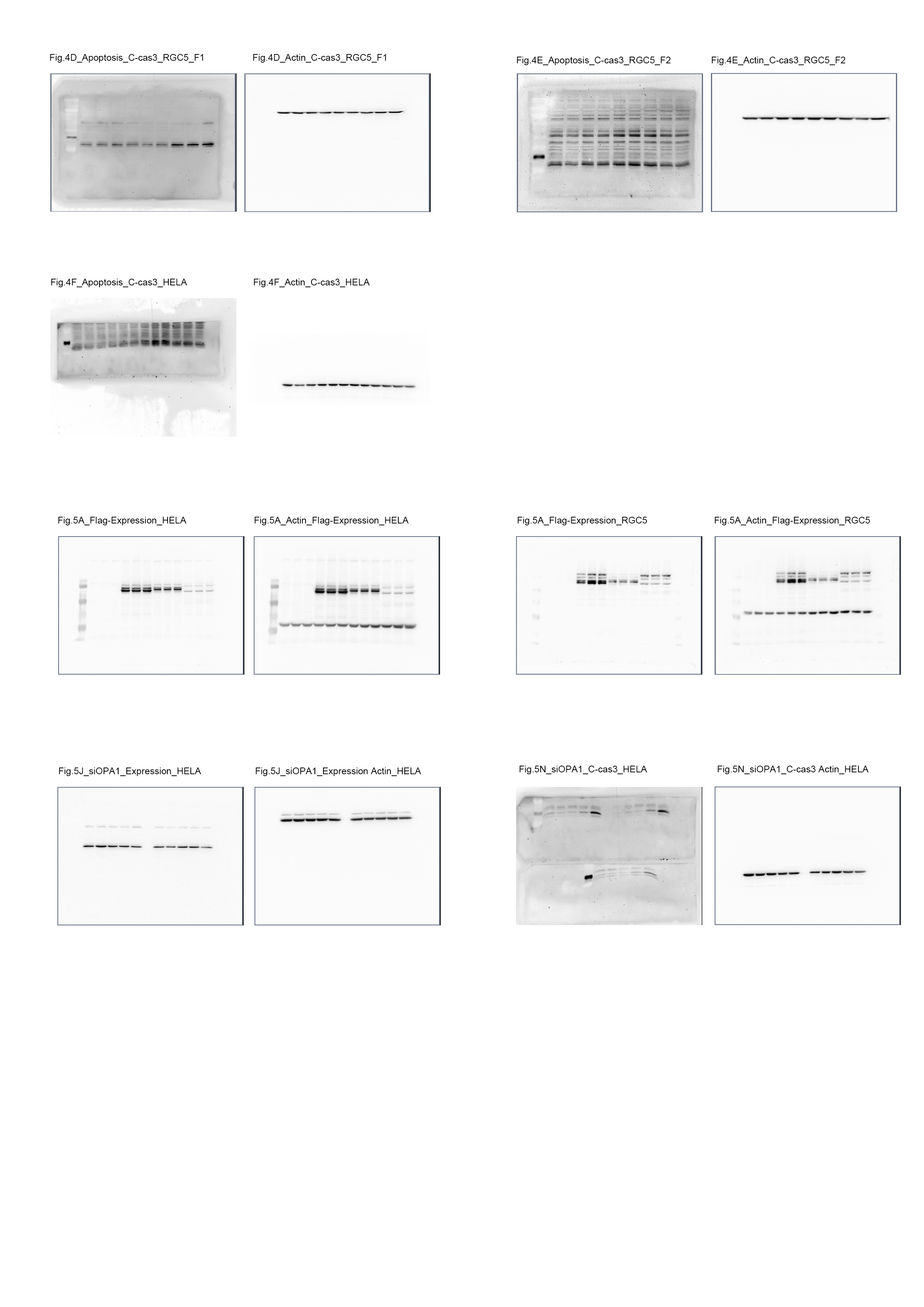

Supplement: Supplementary file 7 — Original WB [file 41420_2025_2442_MOESM7_ESM.tif]
